# MR-Guided Microwave Ablation in T1 Renal Cell Carcinoma: Initial Results in Clinical Routine

**DOI:** 10.1155/2021/5537192

**Published:** 2021-05-13

**Authors:** Zhaonan Li, De-Chao Jiao, Chaoyan Wang, Wenguang Zhang, Jing Li, Xinwei Han

**Affiliations:** Department of Interventional Radiology, First Affiliated Hospital of Zhengzhou University, Zhengzhou 450000, China

## Abstract

**Objective:**

Percutaneous tumor ablation is usually performed using computed tomography (CT) or ultrasound (US) guidance, although reliable visualization of the target tumor could be challenging. Magnetic resonance- (MR-) guided ablation provides more reliable visualization of the target tumors and allows multiplanar imaging of the treatment process, making it the modality of choice, in particular if lesions are small.

**Methods:**

From March 2016 to January 2018, 32 patients scheduled for percutaneous treatment of T1 RCC underwent MR-guided MWA. Complications were classified according to the Clavien grade. Kaplan–Meier survival estimates were calculated to evaluate progression-free survival (PFS).

**Results:**

Technical success was achieved in all lesions. The mean energy and procedure duration were 61.6 ± 8.7 kJ and 118.2 ± 26.7 min, respectively. The glomerular filtration rate (GFR) dropped rapidly after 1 month of treatment and slowly recovered within three months (*P* < 0.05). Postoperative pain and fever were the most common adverse events after treatment. Perirenal hematoma, thermal injury of the psoas muscle, and abdominal distension were common complications after MWA, and the incidence rates were 9.4% (3/32), 6.3% (2/32), and 6.3% (2/32), respectively. According to the Clavien grade classification, serious complications include hydrothorax, bowel injury, and renal failure, all of which have a probability of 3.1%. Of note, the three serious complications occurred in one patient. The 1-, 2-, and 3-year PFS rates were 96.9%, 93.8%, and 83.9%, respectively. The mean PFS rates were 33.972 months (95% CI: 33.045, 35.900).

**Conclusion:**

Microwave ablation is feasible under MR guidance and provides effective treatment of RCC in one session.

## 1. Introduction

Renal cell carcinoma (RCC) has a high malignant tendency and usually asymptomatic or presents with vague symptoms, making an early diagnosis while still a localized disease a challenge [1, 2]. Due to the decrease in morbidity and the increase in renal function retention, partial nephrectomy (PN) may be a beneficial choice for patients with T1 RCC. However, recent studies have revealed that thermal ablation versus RN or PN for T1a RCC results in almost 100% cancer-specific survival (CSS) at 7 yr for all three treatment groups [3]. In fact, thermal ablation is a maturing treatment option for RCC, which provides an even more safe, effective, and minimally invasive option compared to what PN offers. According to The European Association of Urology (EAU), the thermal ablation of renal cancer is a recognized option for patients with suboptimal surgery [4]. In comparison with radiofrequency ablation (RFA) or cryoablation (CA), MWA has the advantage of producing a greater amount of ablation in a shorter period of time and significantly reducing the perivascular heat sinking effect [5, 6]. Recently, magnetic resonance imaging (MRI) is a promising guiding method for MWA, which has the advantages of arbitrary direction imaging, high tissue resolution, and unique temperature sensitivity. The purpose of this study was to evaluate the feasibility and the safety of MRI-guided and monitored MWA of RCC.

## 2. Materials and Methods

### 2.1. Patients

**Figure 1:**
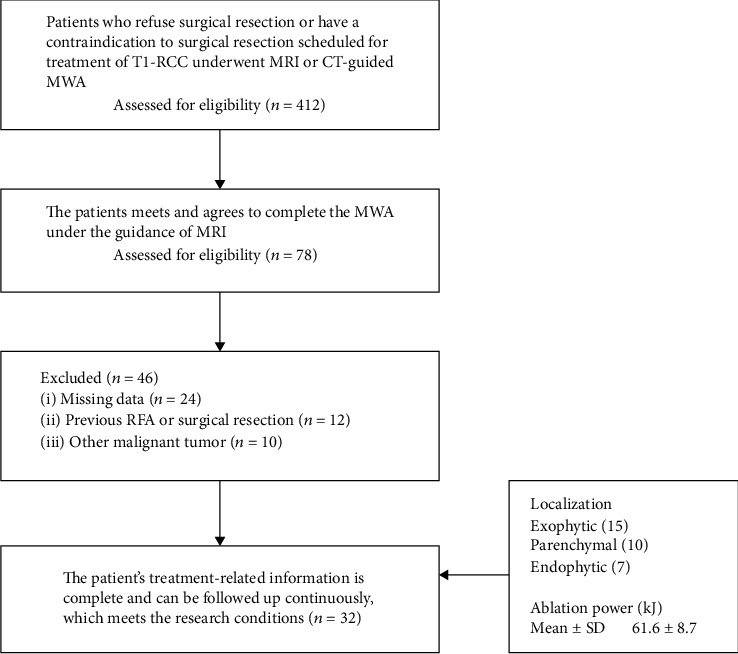
The treatment flowchart.

The institutional review board of our hospital approved the retrospective study, and written informed consent was obtained from each patient. All procedures performed in studies involving human participants were in accordance with the ethical standards of the institutional and/or national research committee and with the 1964 Helsinki declaration and its later amendments or comparable ethical standards. Between March 2016 and January 2018, we retrospectively enrolled 32 patients in our institution who received MR-guided MWA of T1 RCC (51.8 ± 10.8 years; range 36–71 years). The pathological types of RCC were clear cell carcinoma (18/56.3%), papillary carcinoma (6/18.8%), clear cell with pseudopapillary features (3/9.4%), and tubule-papillary carcinoma (3/9.4%), respectively. The inclusion and exclusion criteria are shown in Table 1. The detailed treatment flowchart is shown in (Figure 1).

### 2.2. Equipment

In this study, the microwave probe (ECO-100AI13, 1.8 mm, 15 cm, Co., Ltd. Nanjing, China) and MR-compatible MWA apparatus (2450 MHz, ECO Medical Instrument Co., Ltd. Nanjing, China) were placed at a distance of 2.5-3 meters beside the MR-compatible operating table. ECG gating sensors and respiratory were placed around the finger and chest wall, respectively. After using the cod liver oil capsule matrix to mark the surface of the body, a standard MR protocol was completed to locate intrahepatic lesions. The procedure was guided by a 3.0 T dual gradient MRI (Magnetom Verio 3.0 T scanner, Siemens Healthineers, Germany) with a 70 cm inner-diameter closed bore. The MR scan sequence and parameters used in our study are shown in (Table 2):

### 2.3. Ablation Procedure Protocol

All patients used general anesthesia to assist the treatment process. All procedures were performed alternately by two interventional radiologists with 5-10 years of experience in ablation. A suitable position was chosen according to the location of the tumor to complete the MWA process. First, a set of cod liver oil capsules was placed on the skin as a guide for applicator placement and a T1WI sequence to determine the puncture path was performed, which was as short as possible and avoids important structures. The puncture site was sterilized, and 2% lidocaine was used for local anesthesia. Under the guidance of MR (T1WI VIBE, 16 s), a microwave applicator was inserted into the center of the tumor and multiple scans were performed to confirm that the applicator tip is 0.5–1 cm beyond the distal tumor. At the same time, the tumor was ablated at 50-70 W for 4-7 minutes. During ablation, a series of monitoring T2 rapid (HASTE, 16 s) and T1 (VIBE, 16 s) sequences were performed continuously, and the ablation range was monitored every 16 s. If the MR indicates that the ablation area did not cover 110% of the lesion, the applicator was requisitioned and multiple overlapping ablations were needed. After the operation, we pulled out the applicator and referred the patient to the ward to observe the postoperative changes closely.

### 2.4. Follow-Up

The follow-up included routine physical examination, laboratory examination (hepatic and renal function tests, blood routine, coagulation function tests) at 1 month and 3 months after treatment, and ultrasound, CT, or MR-enhanced scans were performed. Then, follow-up examinations are conducted every six months. Follow-up was closed at the time of death or disconnected.

## 3. Results

### 3.1. Patient Characteristics and Changes in Renal Function

The mean age of the patients was 54.8 ± 8.2 years (range, 39–67 years). Of the 32 patients, 20 (62.5%) were male, 18 (56.3%) had RCC at the T1a stage, and the rest had T1b stage (Table 3). Among the 32 patients, endophytic, parenchymal, and central RCCs were 46.9% (15/32), 31.3% (10/32), and 21.8% (7/32), respectively. Technical success was achieved in all lesions, and no recurring tumor was detected 1 month after the procedure by CT or MRI examination. The mean energy and procedure duration were 61.6 ± 8.7 kJ and 118.2 ± 26.7 min, respectively. The hospitalization length after the intervention was 1.7 ± 1.8 days (range: 1-6 days).

### 3.2. MRI Findings and Technical Success

**Figure 2:**
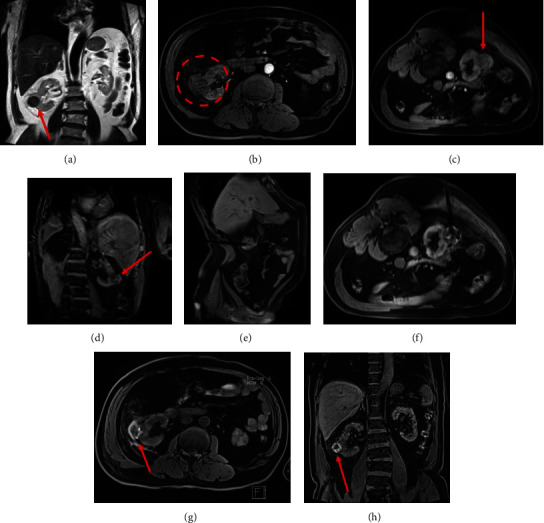
The procedure of treatment of a 54-year-old male patient with T1 renal cell carcinoma. When the patient uses T2 rapid (HASTE, 16 s) and T1 (VIBE, 16 s) sequences supine scans, it is found that the tumor is located on the right side of the body ((a) red arrow, (b) dashed circle). The lateral ablation angle cannot be implemented in the closed MRI modality, so the patient's position needs to be adjusted to find the best ablation path. Adjust the patient's body position to the oblique position ((c, d) red arrow), precisely insert the applicator into the target lesion position under the guidance of the continuous T1 (VIBE, 16 s) sequence, and ablate the lesion in the transverse position (e, f). The MWA-induced damage zone estimated as hyperintensity on T1 high signal range completely covers the tumor after during ablation ((g, h); red arrow).

Before MWA, the tumor showed high intensity on T2WI (HASTE, 16 s) and low signal on T1WI (VIBE, 16 s) (Figures 2(a) and 2(b)). Additionally, the relationship between the tumor and the surrounding tissues can be displayed in the lateral and oblique positions through multiplane imaging (Figures 2(c) and 2(d)). The microwave antenna appeared as a low-signal intensity band on each MR sequence (Figures 2(e) and 2(f)). As the ablation time of the T1WI (VIBE, 16 s) sequence was monitored, the signal intensity of the tumor gradually increased. Thus, the appearance of normal hepatic tissue surrounding the spherical high-signal ablation zone constituted a typical ‘target sign' (Figures 2(g) and 2(h)). When the tumor is close to a specific location (near the intestine and abdominal wall), the ablation needle needs to be adjusted slowly under the guidance of the T1WI sequence to avoid tissue damage. Some patients need to change the patient's normal position to choose an oblique position to assist puncture and complete ablation treatment. All patients with MR-guided MWA achieved technical success and technical effectiveness.

### 3.3. Changes in Renal Function

Changes in renal function were evaluated in all 32 patients. Mean baseline estimated GFR were (74.56 mL/min/1.73 m^2^ ± 23.4; range 21.9-112.1 mL/min/1.73 m^2^). Mean GFR was reduced considerably compared the baseline level with 1 month (62.0 mL/min/1.73 m2 ± 21.0; range, 27.8–108.6 mL/min/1.73 m^2^; *P* = 0.016), 3 months after MWA (66.9 mL/min/1.73 m^2^ ± 19.5; range, 33.8–110.1 mL/min/1.73 m^2^; *P* = 0.033). The median pre-MWA creatinine level was 0.92 mg/dL, which significantly increased to 1.06 mg/dL after 3 months of treatment (*P* = 0.344) (Table 4).

### 3.4. Complications and PFS

**Figure 3:**
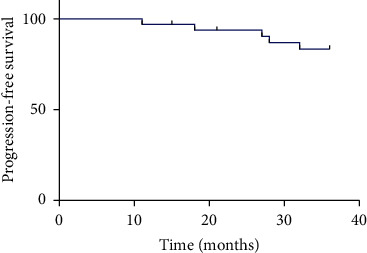
Kaplan–Meier progression-free survival (PFS) with MR-guided treatment. The 1-, 2-, and 3-year PFS rates were 96.9%, 93.8%, and 83.9%, respectively. Mean PFS rate was 33.972 months (95% CI: 33.045, 35.900) .

Postoperative pain and fever were the most common adverse events after treatment. Perirenal hematoma, thermal injury of the psoas muscle, and abdominal distension were common complications after MWA, and the incidence rates were 9.4% (3/32), 6.3% (2/32) and 6.3% (2/32), respectively. According to the Clavien grade classification, serious complications include hydrothorax, bowel injury, and renal failure, all of which have a probability of 3.1%. Of note, the three serious complications occurred in one patient (Table 5). The 1-, 2-, and 3-year PFS rates were 96.9%, 93.8%, and 83.9%, respectively. The mean PFS rates were 33.972 months (95% CI: 33.045, 35.900) (Figure 3). Two patients underwent radical nephrectomy, and the rest received additional MWA.

## 4. Discussion

MWA is increasingly used in the treatment of RCC and has achieved certain clinical results [7, 8]. In a propensity-matched cohort study of 1955 patients with percutaneous MWA and laparoscopic PN for the treatment of cT1a RCC, it was found that there were no significant differences regarding oncologic outcomes and complications between the two therapies [9]. Furthermore, percutaneous MWA leads to smaller changes in renal function and lower blood loss. For patients who cannot accept the risk of more invasive laparoscopic PN, percutaneous MWA could be an alternative less invasive treatment option. The evidence of MWA in RCC indicates that MWA has a similar or slightly higher technical efficacy rate and local tumor control rate than RFA or CA, and the incidence of major complications after MWA is relatively low among the three options [10]. A meta-analysis showed that percutaneous RFA and CA were equally effective and safer than surgical treatments [11]. Moreland et al. studied 66 patients with RCC who received high-power MWA and found that all patients met the expected treatment standard and showed stability of eGFR in the short term [12]. In fact, the high perfusion and potential heat dissipation of the kidney may alter the bioheat equation, leading to poor local tumor control. Of note, the kidney has almost 4 times high perfusion than that of the liver, and its unique physiological characteristics require a more precise treatment method for the current ablation. MWA can produce a larger ablation volume in a shorter time and significantly reduce the perivascular heat sinking effect around blood vessels. Recently, the use of a reasonable and effective imaging modality is also vital for a successful and safe ablation. Because MRI provides the most reliable visualization of target tumors, superior soft tissue contrast, and ability to perform multiplane imaging [13, 14], MRI is considered a promising modality to guide the MWA procedure.

As the procedure is limited by the magnetic compatibility of the microwave ablation kit and the high cost of treatment, there are few clinical reports on a MR-guided thermal ablation for RCC. Andreas et al. [15, 16]. reported MR-guided RFA for RCC in 2005 and 2008, respectively, showed a high technical success rate and favorable clinical efficacy. Recently, MR-guided ablation has been gradually applied to the minimally invasive treatment of primary and metastatic renal malignancies [17, 18]. The MR-guided method could ensure precise insertion of the applicator into the target tissue and enable monitoring and controlling the degree of tissue coagulation, to confirm that the tumor has been completely destroyed as well as reduce the impact on normal renal tissue and thermal damage to adjacent structures [19–22]. The characteristic of coagulative necrosis during MR-guided ablation is that the signal intensity on T2WI decreases while the signal intensity on T1WI increases. Actually, the changes in the signal of the tumor are closely related to the water content of the tissue. The ablation process significantly reduces the water content in the tissue, contributing to a reduction in both T1 and T2 values, and the lesions appear hyperintense on T1WI and hypointense on T2WI with clear, well-defined edges.

During the study period, we changed the anesthesia method that used local anesthesia under CBCT-guided MWA from conscious sedation and local anesthesia to general anesthesia to control pain and related adverse reactions [23, 24]. We found that sedation and analgesia alone cannot keep the patient in the treatment position for a long time, and the claustrophobic environment of the MRI room may increase the psychological burden of the patient. Second, research also showed that many tumors need a specific body position for the best puncture path determination, due to their tumor's location. As for our cases, the kidneys were located on both sides of the body and the best puncture path was commonly affected by the closed-loop magnetic resonance equipment. When the conventional supine position cannot meet the needs of the ablation path, choosing an inclined lateral position may increase the operating space during the procedure, reducing the number of punctures thereby increasing the ablation rate. However, in some of our study cases, because the pain during the procedure cannot be controlled by the use of local sedation and analgesia, the patient's inability to continue the treatment process led to incomplete ablation of the tumor margin and local tumor progression. Therefore, further research is necessary to evaluate how general anesthesia affects the outcome of MR-guided MWA treatment.

In the course of this study, the incidence of complications may be higher than other treatments, which may be related to our statistical methods. If a patient has three different complications, this study will separately count the occurrence probability of each complication, which also causes the actual high complication. In fact, most of the complications of patients are in Clavien grade-I, that is, patients do not need drugs, surgery, endoscopic, and radiological intervention clinical symptoms. Additionally, the PFS rates of T1 RCC under MRI-guided MWA for 1-, 2-, and 3-year were 96.9%, 93.8%, and 83.9%, respectively. The mean PFS rate was 33.972 months (95% CI: 33.045, 35.900). Klapperich et al. performed MWA of 100 cases of T1a stage RCC confirmed by biopsy and found that the 3-year PFS rate was 88%, and the complications were all within a controllable range [25]. The study further confirms that MWA under the guidance of MRI is a safe and effective treatment for stage T1 RCC.

The following limitations need to be addressed. Our study is a retrospective study. Without the design for the comparison with the procedures guided by other modalities such as CT or US, it is worth noting that MRI is the most complicated procedure. Secondly, our study uses closed MRI; although this procedure can ensure high field strength, rapid imaging, and precise targeting, the narrow space limits the best ablation path for some tumors, and the puncture angle of the site needs to be obtained by adjusting the patient's position. Besides, temperature measurement software is not used to monitor the ablation process in real time, which may affect the ablation results. Finally, the study included only 32 patients with RCC and had a short follow-up duration. However, the study's purpose was to investigate the feasibility of MRI-guided and monitored the MWA of RCC. In future studies, larger sample size and longer follow-up time will be required to explore the clinical significance of this technology.

In conclusion, MR-guided percutaneous MWA of RCC is a feasible approach that can be performed under conventional MR guidance. And it was monitored with favorable curative effects.

## Figures and Tables

**Table 1:** Inclusion and exclusion criteria.

Inclusion criteria	Exclusion criteria
1 Age range: 18–75 years	Age < 18 or >75 years
2 T1 renal tumor (≤4 cm)	Non-T1 stage renal tumor (≥4 cm)
3 ECOG score≦2	ECOG score ≥ 2
4 PLT ≥ 40 × 10^9^/L or PT ≤ 25 s	PLT ≤ 40 × 10^9^/L or PT > 25 s
5 The expected survival time > 3 months	The expected survival time ≤ 3 months
6 Patients had a single RCC	Patients had multiple RCC
7 None had hereditary RCC	Patients had hereditary RCC
8 Have a contraindication to surgical resection or refuse to undergo surgical resection	

ECOG: Eastern Cooperative Oncology Group; PLT: platelet; PT: prothrombin time; RCC: renal cell carcinoma.

**Table 2:** The MR scanning sequences and parameters.

Section	Sequence	TE(ms)	TR (ms)	Slice thickness (mm)	Matrix	Flip angle	Band width (Hz/pixel)
Transverse section	T1 VIBE	1.93	4.56	3.3	216 × 288	9.0	400
Transverse section	T2 HASTE	106	1000	4.5	137 × 256	180	781
Transverse section	Diffusion	83	7100	5.0	192 × 144	90	1670
Coronal section	T1 VIBE	2.46	6.11	3.0	179 × 256	9.0	410
Sagittal	T2 HASTE	106	1000	4.0	137 × 256	180	781

**Table 3:** Patient characteristics.

Characteristics	Patients (*n* = 32)	Percentage (%)
Age (y)		
Mean ± SD	54.8 ± 8.2	
Range	39-67	
Gender		
Male	20	62.5
Female	12	37.5
Comorbid disease		
Yes	19	59.4
No	13	40.6
T stage		
T1a	18	56.3
T1b	14	43.6
ASA score		
Mean ± SD	2.6 ± 0.9	
Range	1-3	
Localization		
Exophytic	15	46.9
Parenchymal	10	31.3
Endophytic	7	21.8
RCC histology		
Clear cell	18	56.3
Papillary	6	18.8
Clear cell with pseudopapillary features	3	9.4
Tubulopapillary carcinoma	3	9.4
Unknown	2	6.1
Ablation power (kJ)		
Mean ± SD	61.6 ± 8.7	
Range	50-70	
Duration (min)		
Mean ± SD	118.2 ± 26.7	
Range	96-162	
Hospitalization		
Mean ± SD	1.7 ± 1.8	
Range	1-6	

**Table 4:** Renal function changes before and after MWA.

Renal function tests	Renal function change			*P*
	M0	M1	M3	
Creatinine (mg/dL)	0.92 (0.55-4.63)	0.98 (0.51-4.93)	1.06 (0.62-6.01)	>0.05
GFR (mL/min/1.73 m^2^)	74.56 (21.9-112.1)	62.07 (27.8-108.6)	66.9 (33.8-110.1)	<0.05

Note—Parenthesis indicates data range. M0: pretreatment; M1: the first month after treatment; M3: the third month after treatment; GFR: glomerular filtration rate; MWA: microwave ablation.

**Table 5:** Summary of complications.

Complications	*N*	Clavien grade
Perirenal hematoma	3 (9.4)	I
Thermal injury of psoas muscle	2 (6.3)	I
Thermal injury of pelvicalyceal system	1 (3.1)	I
Pneumothorax	1 (3.1)	I
Diarrhea	1 (3.1)	II
Abdominal distension	2 (6.3)	II
Edema of lower limbs	1 (3.1)	II
Hydrothorax	1 (3.1)	IIIa
Bowel injury	1 (3.1)	IIIb
Renal failure	1 (3.1)	IVa

Note—Unless indicated, data are numbers of patients, and numbers in parentheses are percentages.

## Data Availability

The clinical data were obtained from the interventional department of the First Affiliated Hospital of Zhengzhou University. The data used to support the findings of this study are available from the corresponding author upon request.
